# Cervical disc arthroplasty with systematic total bilateral uncuscectomy - Adapted technique particularly in severe spondylosis: A prospective study

**DOI:** 10.1016/j.bas.2023.101734

**Published:** 2023-04-01

**Authors:** Henri-Benjamin Pouleau, Olivier De Witte, Alexandre Jodaïtis

**Affiliations:** a Department of Neurosurgery, University Hospital Center Tivoli, La Louvière, Belgium; b Department of Neurosurgery, Academic Hospital Center Erasme, Bruxelles, Belgium

**Keywords:** Uncuscectomy, Uncinectomy, Cervical disc arthroplasty, Cervical prosthesis, Severe spondylosis

## Abstract

**Introduction:**

Cervical disc arthroplasty (CDA) is mainly used in young patients with soft herniated discs and seems to have several advantages over anterior cervical discectomy and fusion (ACDF). Severe spondylosis is common and represents a contraindication for performing CDA.

**Research question:**

Is it possible to expand the indications for the implantation of cervical prostheses by adapting the surgical technique, particularly for severe spondylosis, to benefit from the advantages of prostheses over ACDF ?

**Materials and methods:**

We propose a prospective two-center study to compare the possible clinical benefit of the placement of a cervical prosthesis with systematic total bilateral uncuscectomy (or uncinectomy) compared to the classical technique of ACDF, particularly for severe spondylosis. Visual analog scales for brachialgia, cervicalgia, and neck disability index were measured before and one year after surgery. Odom’s criteria were assessed one year after surgery.

**Results:**

We compared 81 patients treated with CDA and systematic total bilateral uncuscectomy versus 42 patients treated with ACDF for symptomatic radicular or medullary compression. Patients treated with CDA and uncuscectomy showed greater improvements in VASb, VASc, NDI, and Odom’s criteria than those treated with ACDF, with statistically significant results. Moreover, no difference was found between the severe spondylosis subgroup and the non-severe spondylosis subgroup treated with CDA and uncuscectomy.

**Discussion and conclusion:**

This study assessed the value of systematic total bilateral uncuscectomy for cervical arthroplasty. Our prospective clinical results suggest a surgical technique to reduce cervical pain and improve function one year after surgery, even in cases of severe spondylosis.

## Highlights

• Severe spondylosis is common and represents a contraindication for performing cervical disc arthroplasty.
• Expand the indications of cervical arthroplasty by adapting the surgical technique, particularly for severe spondylosis.
• Evaluate clinical improvement in CDA with systematic total bilateral uncuscectomy compared to classical ACDF.

## 1 Introduction

The anterior cervical discectomy and fusion for single-level disc disease was first described by Smith, Cloward, and Robinson in 1958 (Cloward, 1958). This technique is used to relieve mechanical pressure on the spinal nerve roots or spinal cord associated with symptoms refractory to nonsurgical treatment. Since then, anterior cervical discectomy and fusion (ACDF) has become a standard intervention in spinal surgery.

Typical symptoms include radicular pain, weakness, numbness, and difficulty walking (O'Brien et al., 2019). Cervical radiculopathy or myelopathy can be secondary to disc herniation, anterior osteophyte complexes, or bony spurs that cause spinal canal narrowing, spinal cord compression, or nerve root impingement (Bohlman et al., 1993). In addition, ACDF may be helpful in patients presenting with spondylitic radiculopathy. It may be successfully used in patients with both single-and multilevel cervical diseases (Bohlman et al., 1993).

Due to concerns regarding the kinematic and biomechanical issues inherent to fusion of the cervical motion segment, investigators have developed cervical disc arthroplasty (CDA). Maintenance of normal spinal kinematics is a primary goal of CDA. The cervical spine is inherently dynamic, with flexion, extension, and lateral bending in addition to anterior and posterior translation (O'Brien et al., 2019). The first artificial cervical disc replacement was a ball-and-socket design (Cummins et al., 1998), and Rousseau et al. (2008) examined the intervertebral kinematics after the use of this kind of cervical prosthesis, either Prestige LP (Medtronic) or ProDisc-C (Synthes Spine), and concluded that this design did not fully preserve the natural range of motion or center of motion between flexion and extension. This may be attributed to the absence of translation when using a constrained prosthesis (Barrey et al., 2009; Sang et al., 2020). Different devices, such as Mobi-C (LDR Zimmer Biomed), permit restoration of the natural motion of the cervical spine. The mobile bearing translated to the inferior endplate, allowing flexion, extension, and lateral bending. Different sizes of MobiC, up to 19 ​mm wide, exist and allow wide coverage of the endplates even during uncuscectomy (or uncinectomy). Owing to its design, MobiC was the only prosthesis used for CDA in our study.

Currently, CDA is primarily used in young patients with soft disc herniations. Contraindications include severe disc degeneration and spondylosis, severe facet joint degeneration, trauma, tumor, infection, and allergy to materials. Severe spondylosis is defined as bridging osteophytes, loss of disc height greater than 50%, or absence of motion (less than 2° - corresponding to grade IV McAfee classification) (Cummins et al., 1998; Rousseau et al., 2008).

We assume that it would be possible to expand the indications for the implantation of cervical prostheses by adapting the surgical technique, particularly for severe spondylosis, to benefit from the advantages of prostheses over ACDF. Therefore, we propose a prospective bicentric study to compare the possible clinical benefit of the placement of a cervical prosthesis with a systematic total bilateral uncuscectomy compared to the classic technique of ACDF.

## 2 Materiel and methods

### 2.1 Arthroplasty with systematic total bilateral uncuscectomy - surgical technique (Fig. 1)

#### 2.1.1 Patient positioning

A proper implant position is vital for optimal CDA function. The patient is placed in the supine position with support behind the neck. The neck is required to be in a neutral position. We recommend using an OARM (Medtronic) to easily obtain AP and lateral radiographs during the procedure. This allows three-dimensional (3D) imaging at the end of the surgery to confirm perfect positioning. The patient's head is secured with tape over the forehead, chin, and above the lips to ensure that the position is maintained. The surgical level is identified and marked.

#### 2.1.2 Approach

The standard Smith-Robinson approach to the anterior cervical spine is used with a horizontal incision in the skinfold. We find our predicted level and mark it with a bayonet needle that can be confirmed radiographically. Once the level is confirmed, we elevate the longus colli muscles so that the retractors can be placed underneath. We insert Caspar pins, X-rays confirms good positioning (parallel to the endplates) and distract them.

#### 2.1.3 Discectomy

The discectomy is performed under a microscope. We expose the posterior longitudinal ligament and medial part of the two uncus. We systematically mill the uncus until a thin layer of bone like eggshell is obtained; therefore, we recommend using a 4 ​mm diamond bur. The thin residual layer is carefully removed using a curette or a hook. The posterior longitudinal ligament is then opened. The endplates are prepared by gently drilling the surface.

Note that classic ACDF does not include a total bilateral uncuscectomy. In general practice, the surgeon ensures the sufficient opening of the foramen and therefore the release of the root by passing a hook in the foramen.

#### 2.1.4 Implant insertion

The widest and deepest implants must be inserted under radiographic guidance. The prosthesis must be neither too thin nor too thick to avoid migration or facet overload. When the prosthesis is in place, we confirm perfect positioning (lateral and AP) using 3D imaging.

We pay particular attention to hemostasis, and always left a suction drain in place.

### 2.2 Recruitment

After obtaining approval from the ethics committees, we compared the usual management of the same indications by two different surgical teams. We prospectively recruited patients who underwent surgery using the classic ACDF technique without uncuscectomy in a first academic hospital and those who underwent CDA with systematic total bilateral uncuscectomy in a second university hospital between first April 2018 and first April 2020. The inclusion criteria were symptomatic radicular or medullary compression with symptoms refractory to nonsurgical treatment. Note that patients with only cervical pain were excluded from the study. The exclusion criteria were severe facet joint degeneration at the surgical level, trauma, tumor, infection, allergy to materials, hybrid construction (CDA and ACDF), anterior and posterior approaches, and revision of the surgical level. Patients with severe disc degeneration or spondylosis were not excluded from the study. Assessment of the condition of the cervical facets was left to the surgeon's discretion based on a cervical CT scan.

Visual analog scales for brachialgia (VASb), cervicalgia (VASc), and the neck disability index (NDI) were measured before and one year after surgery. Odom's criteria were assessed one year after surgery.

We used the Wilk-Shapiro, Mann-Whitney, and Wilcoxon signed-rank tests for the statistical analyses.

## 3 Results

63 patients meeting the inclusion and exclusion criteria underwent ACDF, and 117 patients underwent CDA with total bilateral uncuscectomy. 47 patients in the ACDF group and 91 patients in the CDA group agreed to participate in the study. Four patients in the ACDF group and 10 patients in the CDA group were lost to follow-up. One patient who underwent ACDF required a second intervention to remove the material less than a year after the first operation because of arthrodesis instability. The average age at the time of surgery was 50 (±9,7) years in the ACDF group and 50,5 (±9,7) years in the CDA group. The male-to-female ratio was similar between the groups. 32 patients received one-level ACDF, and 10 patients underwent two-level ACDF for a total of 52 fused levels. 29 patients received one-level CDA, 42 patients received two-level CDA, and 10 patients received three-level CDA, for a total of 143 levels treated (Fig. 2).

**Fig. 1:**
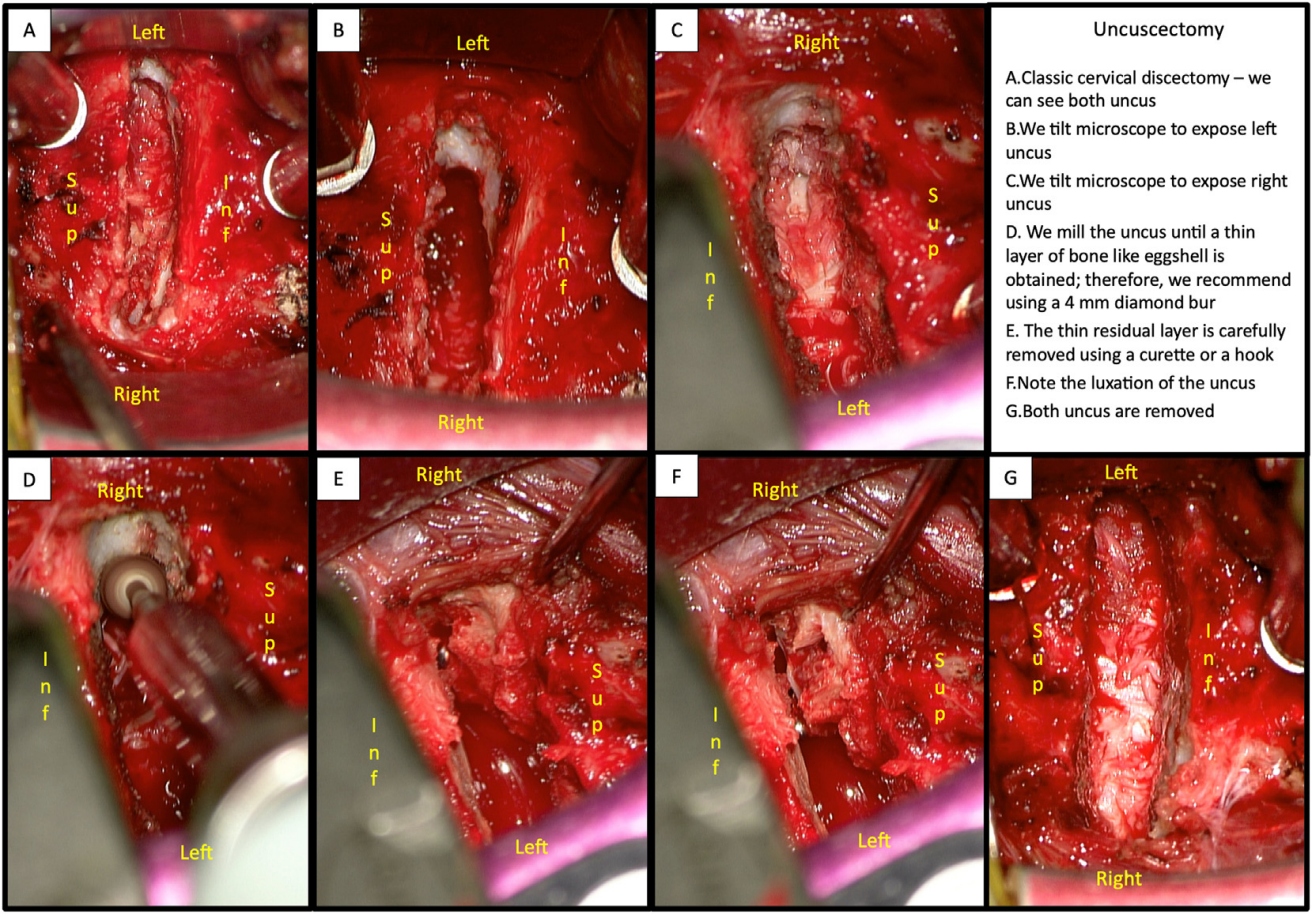
Uncuscectomy A. Classic cervical discectomy - we can see both uncus B. We tilt microscope to expose left uncus C. We tilt microscope to expose right uncus D. We mill the uncus until a thin layer of bone like eggshell is obtained; therefore, we recommend using a 4 ​mm diamond bur E. The thin residual layer is carefully removed using a curette or a hook F. Note the luxation of the uncus G. Both uncus are removed.

**Fig. 2:**
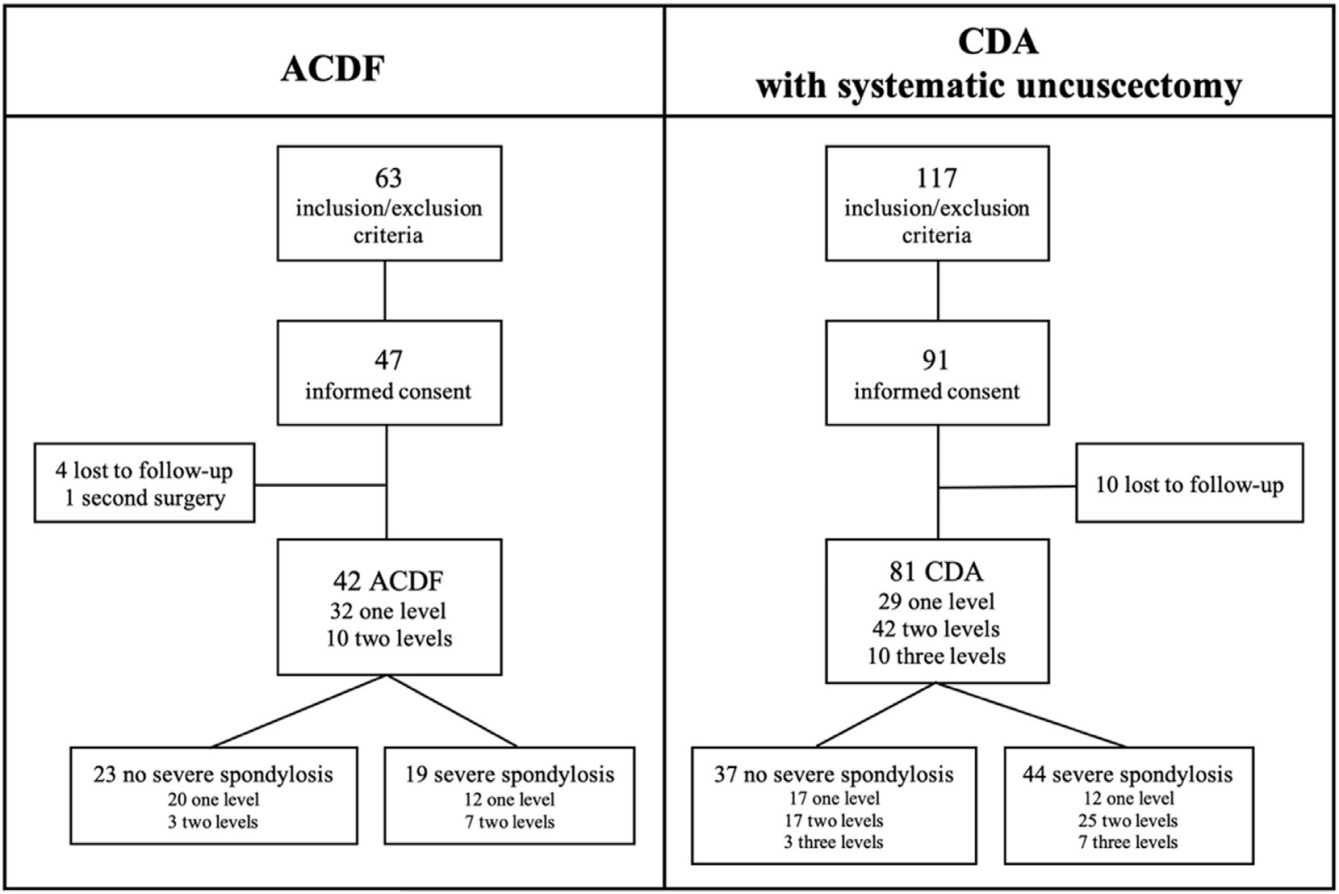
Recruitment.

The median preoperative VASb was 8,0 [6; 9] for ACDF and 8,0 [7; 9] for CDA and decreased to 1,5 [0; 6] (p<0,0001) and 0 [0; 3] (p<0,0001) respectively one year after the surgery. The median preoperative VASc was 7,5 [4,75; 9] for ACDF and 8,0 [7; 10] for CDA and decreased to 3,5 [1,25; 7] (p ​= ​0,001) and 2 [0; 4] (p<0,0001) respectively. ΔVASb was −4,0 [−7;-1] in the ACDF group and −7,0 [−8;-4] in the CDA group (p ​= ​0,003) and ΔVASc was −2,0 [−4; 0] in the ACDF group and −6,0 [−8;-3] in the CDA group (p<0,0001). The median preoperative NDI was 20,5 [13,25; 26,75] for ACDF and 28,0 [23; 35] for CDA and decreased to 10 [4; 25,75] (p ​= ​0,024) and 3 [0; 6] (p<0,0001) respectively. ΔNDI were −2,5 [−14; 3] in the ACDF group and −24,0 [−30;-19] in the CDA group (p<0,0001). Odom's criteria were 2 [2; 3] (good) for ACDF and 1 [1; 2] (excellent) for CDA (p<0,0001).

Improvements in the VASc and NDI for two-level ACDF were not statistically significant in our study. Postoperative VASb, VASc and NDI were better regardless of the number of levels treated by CDA with statistically significative results (Table 1).

**Table 1:** Preoperative and 1 year postoperative VASb, VASc and NDI.

	Male/	Age	Median VAS brachialgia		Median VAS cervicalgia		Median NDI	
	Female		Preoperative	1 year postoperative	Preoperative	1 year postoperative	Preoperative	1 year postoperative
ACDF (42)	19/23	50 (±9,7)	8 [6; 9]	1,5 [0; 6]	7,5 [4,75; 9]	3,5 [1,25; 7]	20,5 [13,25; 26,75]	10 [4; 25,75]
			p S (<0,0001)		p S (= 0,001)		p S (= 0,024)	
**One level (32)**	15/17	48,8 (±10,6)	8 [6; 9]	2 [0; 6]	8 [5; 9]	4 [1; 7]	21 [14; 29]	14 [4; 26]
			p S (0,0002)		p S (= 0,001)		p S (= 0,009)	
**Two levels (10)**	4/6	54 (±5,5)	7,5 [7; 8,75]	0,5 [0; 6,5]	7 [4; 8]	4 [3; 7,5]	19 [13,75; 24,75]	10 [6,75; 25,8]
			p S (= 0,018)		p NS (= 0,344)		p NS (= 0,759)	
**No severe spondylosis (23)**	11/12	45,7 (±7,5)	7 [6; 9]	1 [0; 5]	8 [7; 9]	3 [1; 6,5]	20 [14; 27]	7 [2; 25,5]
			p S (= 0,003)		p S (= 0,004)		p NS (= 0,056)	
**Severe spondylosis (19)**	8/11	56 (±9,2)	8 [6; 9]	2 [0; 6]	7 [4; 9]	5 [1,5; 8]	21 [9; 27]	10 [5,5; 26]
			p S (= 0,001)		p NS (= 0,095)		p NS (= 0,248)	

CDA with uncuscectomy (81)	37/44	50,5 (±9,7)	8 [7; 9]	0 [0; 3]	8 [7; 10]	2 [0; 4]	28 [23; 35]	3 [0; 6]
			p S (<0,0001)		p S (<0,0001)		p S (<0,0001)	
**One level (29)**	13/16	47,1 (±9,8)	8 [7; 10]	0 [0; 4]	9 [7; 10]	2 [0; 4]	28 [22; 36]	3 [0; 6]
			p S (<0,0001)		p S (<0,0001)		p S (<0,0001)	
**Two levels (42)**	21/21	52 (±9,7)	8 [7; 9]	0 [0; 2]	8 [7; 9]	1 [0; 2,75]	29 [23; 32]	2 [0; 5]
			p S (<0,0001)		p S (<0,0001)		p S (<0,0001)	
**Three levels (10)**	3/7	54,3 (±6)	8 [7; 9]	2 [0,25; 3,75]	8,5 [6,25; 10]	2 [0; 3,75]	29 [25; 34]	5 [0,5; 7]
			p S (= 0,007)		p S (= 0,005)		p S (= 0,002)	
**No severe spondylosis (37)**	19/18	46,4 (±8,6)	8 [7; 9]	0 [0; 3]	8 [8; 9]	1 [0; 4]	28 [22; 35]	3 [0; 6]
			p S (<0,0001)		p S (<0,0001)		p S (<0,0001)	
**Severe spondylosis (44)**	18/26	54 (±9,2)	8 [7; 9]	0 [0; 2,25]	8 [6,75; 10]	2 [0; 3]	28 [23; 34,25]	3 [0,75; 5,25]
			p S (<0,0001)		p S (<0,0001)		p S (<0,0001)	

**Fig. 3:**
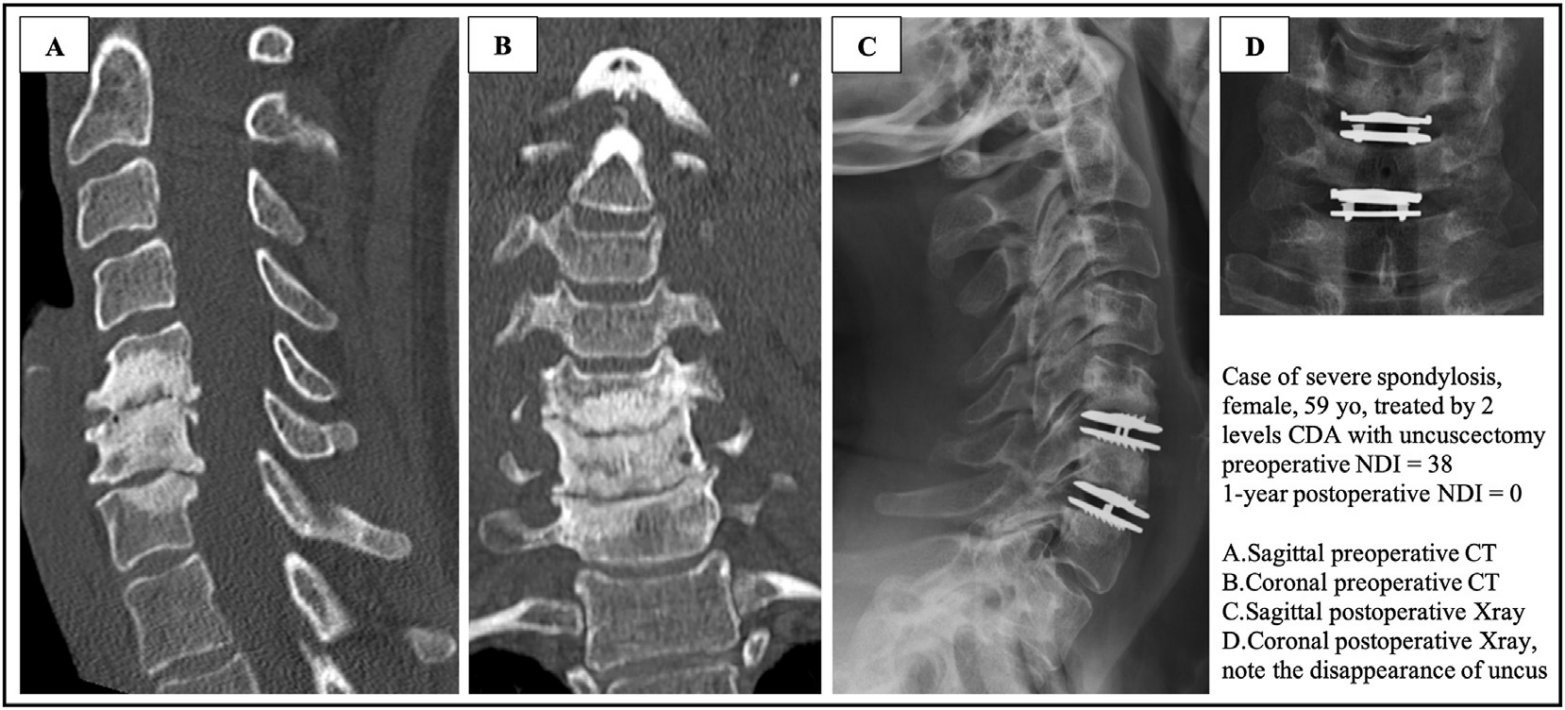
Case of severe spondylosis, female, 59 yo, treated by 2 levels CDA with uncuscectomy; preoperative NDI = 38; 1-year postoperative NDI ​= ​0; A. Sagittal preoperative CT B. Coronal preoperative CT C. Sagittal postoperative Xray D. Coronal postoperative Xray, note the disappearance of uncus.

Nineteen patients treated with ACDF (12 one-level and 7 two-levels) and 44 patients treated with CDA (12 one-level, 25 two-levels and 7 three-levels) had at least one criterion of severe spondylosis (Fig. 3). The NDI and VAS results obtained in the severe spondylosis CDA subgroup were similar to those obtained in the non-severe spondylosis CDA subgroup, and no statistical difference was found. Moreover, patients with severe spondylosis treated with CDA and total bilateral uncuscectomy showed significantly better ΔVASb, ΔVASc, ΔNDI, and Odom's criteria than those treated with ACDF (Table 2).

**Table 2:** Evolution of pain in the ACDF and CDA groups according to severe pre-existing spondylosis.

	Total group		No severe spondylosis		Severe spondylosis	
	ACDF	CDA with uncuscectomy	ACDF	CDA with uncuscectomy	ACDF	CDA with uncuscectomy
Δ VAS brachialgia	−4 [-7;-1]	−7 [-8;-4]	−5 [-7;-0,5]	−7 [-8;-4]	−1 [-6,5;-0,5]	−7 [-8;-3,75]
	p S (= 0,003)		p S (= 0,034)		p S (= 0,017)	
Δ VAS cervicalgia	−2 [-4; 0]	−6 [-8;-3]	−2 [-4; 0]	−5 [-8;-3]	−1 [-4; 0]	−7 [-8;-3875]
	p S (<0,0001)		p S (= 0,001)		p S (= 0,0002)	
Δ NDI	−2,5 [-14; 3]	−24 [-30;-19]	−6 [-14; 3]	−26 [-31;-19]	−2 [-12; 3]	−24 [-29,25;-17,5]
	p S (<0,0001)		p S (<0,0001)		p S (<0,0001)	
Odom's criteria	2 [3; 2] (good)	1 [2; 1] (excellent)	2 [3,5; 1,5] (good)	1 [2; 1] (excellent)	3 [3; 2] (fair)	1 [2; 1] (excellent)
	p S (<0,0001)		p S (= 0,001)		p S (= 0,0003)	

No major intraoperative complications or damage to the vertebral artery occurred in either the CDA or ACDF group. We haven't experienced postoperative Horner syndrome. To our opinion, it's because we lift the longus colli muscle from the median part to the lateral part. We don't cross the muscle and so we avoid the risk of causing Horner syndrome. A few patients have presented spontaneously resolving transient paraesthesias in the upper limbs postoperatively.

## 4 Discussion

Adjacent segment degeneration (ASD) is one of the foremost concerns in ACDF. Several meta-analyses have been carried out in recent years and have attempted to prove the superiority of CDA in avoiding ASD (Luo et al., 2018; Lian et al., 2017; Shriver et al., 2016; Zhang et al., 2020; Wang et al., 2020; Latka et al., 2019). These publications identified a significant increase in ASD with longer follow-up periods. Pseudoarthrosis is another concern associated with ACDF, and it becomes more prevalent as the number of fused segments increases. Pseudoarthrosis has been reported in 11% of single-level fusions and 27% of multilevel fusions (Bohlman et al., 1993).

Because of these concerns as well as the desire to preserve motion and return patients to routine activities, CDA appeared approximately 30 years ago, and many different materials were developed. Every cervical prosthesis has a different conception and, therefore, exhibits a different behavior in the cervical spine. This partly explains the difficulty in drawing reliable conclusions from CDA literature. However, several meta-analyses have shown the superiority of CDA over ACDF in terms of pain improvement (Kan et al., 2016; Ma et al., 2016; Zou et al., 2016). However, classical contraindications, particularly severe spondylosis, have limited the use of cervical prostheses.

The classic ACDF technique and our CDA technique reduced brachialgia and cervicalgia one year after surgery. In our study, patients treated with CDA with systematic total bilateral uncuscectomy showed greater improvements in VASb, VASc, NDI, and Odom's criteria than those treated with ACDF, with statistically significant results. Moreover, no difference was found between the severe and non-severe spondylosis subgroups treated with CDA and total bilateral uncuscectomy. Therefore, this study argues that severe spondylosis should not be a contraindication for CDA if an adapted surgical approach is used. Approximately half of the patients undergoing arthroplasty in our study had severe spondylosis and, in theory, should not have benefited from this technique, while clinical results were better one year postoperatively in the CDA group.

We believe that two points are essential for cervical arthroplasty. First, using a prosthesis with the capacity to restore the natural motion of the cervical spine. Second, allowing the cervical spine to regain its original mobility. This second step is possible because of systematic total bilateral uncuscectomy, particularly in cases of severe spondylosis, provided that the facet joints are in good condition. If the surgeon places a cervical prosthesis in cases of severe spondylosis with the same discectomy technique as for an ACDF, the surgeon does not restore segmental mobility. This is why CDA is normally contraindicated in severe spondylosis. If the surgeon wants to place a cervical prosthesis in severe spondylosis case, he must absolutely adapt the technique to restore mobility.

We believe that restoring mobility of the cervical spine will allow the prosthesis to balance itself according to the natural balance of the cervical spine, thus reducing cervical pain. In a future study, we propose to evaluate the “automatic” restoration of cervical sagittal balance during CDA with total bilateral uncuscectomy.

Uncuscectomy in the context of CDA is not a common technique reported in literature. M. Makhni shares tips and tricks about CDA (Makhni et al., 2019) and seems to share the same interest in uncuscectomy to prevent the reappearance of osteophytes. Long-term studies will allow us to determine whether uncuscectomy can delay reduction in prosthesis mobility.

Moreover, VASb levels were lower in the CDA group. A possible explanation for these results could be linked to the fact that the opening of the foramina is not always sufficient in the ACDF group. If a systematic uncuscectomy is performed, the foramina are completely opened, and the roots are decompressed. Note that uncuscectomy was originally developed because some patients operated by ACDF with severe uncarthrosis did not benefit from sufficient root decompression. Neurological symptoms are then persistent postoperatively. In this context, several studies have shown the interest and feasibility of performing a cervical uncuscectomy during ACDF (Safaee et al., 2020; Chen et al., 2001; Jho, 1996; Johnson et al., 2000; Saringer et al., 2002).

Finally, no major intraoperative complications occurred in either of the groups. Our results are consistent with those of a 2017 FDA meta-analysis showing that CDA appears to be as safe as or safer than ACDF at 2-year follow-up (Anderson et al., 2017).

### 4.1 Limits of the study

One-year follow-up remains relatively short. In addition, this first study of CDA with systematic total bilateral uncuscectomy was essentially focused on the clinical evolution of patients. We are currently carrying out other studies, in particular to analyze radiological results. Moreover, it will be interesting in coming years to observe clinical and radiological results to assess pain evolution, restoration of sagittal balance, appearance of osteophytes, risk of instability in the long-term and adjacent level degeneration.

## 5 Conclusions

To the best of our knowledge, this is the first clinical study to assess the value of systematic total bilateral uncuscectomy in cervical arthroplasty. Our prospective clinical results suggest a surgical technique to reduce cervical pain and improve function one year after surgery, even in cases of severe spondylosis. Additional studies, particularly radiological studies, would be of interest to assess the restoration of sagittal balance, appearance of osteophytes, and risk of instability in the long-term.

## Funding

The authors declare that no funds, grants, or other support were received during the preparation of this manuscript.

## Author contributions

All authors contributed to the study conception and design. Material preparation, data collection and analysis were performed by HB Pouleau. The first draft of the manuscript was written by HB Pouleau and all authors commented on previous versions of the manuscript. All authors read and approved the final manuscript.

## Ethics approval

This study was performed in line with the principles of the Declaration of Helsinki. Approval was granted by the Ethics Committee of Erasme University Bruxelles.

EUDRACT reference: B406201835482.

## Consent to participate

Informed consent was obtained from all individual participants included in the study.

## Consent to publish

The authors affirm that human research participants provided informed consent for publication of the images in Fig. 1.

## Declaration of competing interest

A.Jodaïtis receives royalties (annual payments) from Zimmer-Biomet (conceptor MobiC).

Others authors have no relevant financial or non-financial interests to disclose.
